# Response Changes in Biological Soil Crusts (BSCs) to Different Sand-Fixing Plantations in Alpine Sandy Land

**DOI:** 10.3390/biology15120910

**Published:** 2026-06-10

**Authors:** Xionglian Jin, Feng Qiao, Zhe Chen, Qiaoyu Luo, Shaobo Du, Zhiqiang Dong, Shuang Ji, Huichun Xie, Xiaoping Kong

**Affiliations:** 1 College of Geographical Sciences, Qinghai Normal University, Xining 810008, China; 18997124460@163.com (X.J.);; 2 School of Life Sciences, Qinghai Normal University, Xining 810008, China; 3 Key Laboratory of Medicinal Animal and Plant Resources of Qinghai-Tibetan Plateau, Xining 810008, China; 4 National Forest Ecosystem Research Station on the Southern Slope of Qilian Mountains, Huzhu 810500, China; 5 Xining Botanical Garden, Xining 810008, China

**Keywords:** alpine sandy land, sand-fixing plantations, biological soil crusts (BSCs), bacterial community, physicochemical properties, enzyme activities

## Abstract

Biological soil crusts (BSCs) play key roles in arid, semi-arid regions and ecological marginal habitats. This study focused on four types of sand-fixing plantations established in 1990 in alpine sandy land (*Salix psammophila*, SL; *Caragana korshinskii*, NT; *Salix cheilophila*, WL; *Populus simonii*, XYY). Soil samples were collected from bare sand, algae crusts, and moss crusts. Soil particle size distribution, physicochemical properties, and enzyme activity were determined. Then bacterial communities were analyzed using high-throughput (Illumina) sequencing and the correlations among these three factors were examined. The results showed that: (1) From bare sand to algae and moss crusts, the content of fine particles (clay + silt) gradually increased. (2) Soil water content (SWC), nutrients and enzyme activities increased progressively. (3) In the study area, the dominant bacterial phyla of BSCs included Pseudomonadota, Cyanobacteria, Actinobacteriota and Vibrionota. Principal Coordinates Analysis (PCoA) and Analysis of Similarities (ANOSIM) results showed that BSCs drive the differentiation of bacterial communities during succession, while forest stands influence their spatial distribution. (4) Spearman’s correlation and redundancy analysis (RDA) showed that available phosphorus (AP), alkaline hydrolyzable nitrogen (AN), soil organic matter (SOM), catalase (CAT), pH, soil water content (SWC), and alkaline phosphatase (ALP) are key physicochemical factors shaping the bacterial community structure of BSCs. Mantel’s test confirmed that these variables mediated BSCs’ bacterial community structure. This study elucidates the mechanisms underlying ecological restoration via BSCs and provides a theoretical basis for future restoration efforts in alpine sandy land.

## Simple Summary

Biological soil crusts (BSCs) have different ecological functions in different habitats. In arid deserts and arid-semiarid regions, BSCs help stabilize soil, cycle nutrients, and retain water. In contrast, cyanobacterial crusts on sandstone surfaces of humid tropical tepui mountains mainly serve as indicators. They help researchers reconstruct the formation and evolution of primitive soils. This study investigated BSCs in four types of sand-fixing plantations located on the northeastern Tibetan Plateau, specifically in alpine sandy land. The researchers examined soil characteristics, nutrient conditions, enzyme activities, and bacterial communities associated with different stages of crust development. The results showed that as the crusts developed from bare sand to algae and moss crusts, soil quality, moisture, nutrient accumulation, and bacterial diversity generally increased. Plantations dominated by *Salix cheilophila* showed the greatest nutrient accumulation, whereas *Populus simonii* plantations showed the highest enzyme activities and the most distinctive bacterial communities. Soil water content, Catalase and Alkaline Phosphatase were identified as important factors influencing bacterial community composition. These findings help us better understand how BSCs support ecological restoration. They also provide useful information for choosing vegetation types. This is helpful for restoring degraded sandy ecosystems in alpine sandy land.

## 1. Introduction

Driven by climate change and human activity, desertification continues to intensify, posing a major challenge to global ecological security and sustainable regional development [1]. Vegetation restoration is a key measure for combating desertification and enhancing soil and water conservation [2,3]. Since the 1960s, long-term ecological restoration has significantly increased vegetation coverage in sandified areas [4]. Meanwhile, a large number of biological soil crusts (BSCs) have also naturally developed within the plantations. BSCs are complex surface communities formed when cryptogams (algae, lichen, moss), soil microorganisms (bacteria, fungi, and archaea), and other microscopic organisms bind soil surface particles together through secretions such as mycelia, rhizoids, and polysaccharides [2,3]. Based on their morphological characteristics and biological composition, they can be classified into lichens, algae, and moss crusts [5]. As an integral part of sandy ecosystems, BSCs play important ecological roles, including soil and water conservation, carbon sequestration, and nitrogen fixation [6,7].

Bacterial communities are crucial for the growth and development of BSCs. Studying bacterial community structure and diversity provides insight into the formation and ecological functions of BSCs [8]. Up to now, a large body of literature has explored microbial characteristics of BSCs in arid and semi-arid ecosystems. Previous studies have demonstrated that different vegetation and BSC types shape unique microenvironments by modifying soil physicochemical properties [9]. For example, studies in subtropical deserts have revealed distinct bacterial community compositions in BSCs across grassland, shrubland and woodland habitats [10]. Research on temperate deserts has also confirmed that water content, total carbon, and organic carbon are the primary environmental factors driving changes in the composition of the bacterial community in the BSCs beneath *Sabina vulgaris* shrubs [11]. Long-term studies on sand-fixing vegetation further indicate that BSC coverage, thickness and microbial activity rise with restoration age. These characteristics are strongly associated with under-canopy microenvironments, which in turn facilitate the succession of BSCs and enhance topsoil fertility [12,13]. However, there are prominent gaps in current research. The majority of relevant studies are conducted in temperate and subtropical arid sandy regions, with far less attention paid to bacterial community succession of BSCs in alpine sandy land. Previous studies on alpine sandy land have only analyzed the soil physicochemical properties and microbial composition during the succession of BSCs under different sand-fixing plants [14,15]. There has been a lack of systematic research on long-term vegetation restoration over a 30-year period, and the mechanisms by which different BSCs in planted forests influence bacterial communities have not yet been elucidated. At the same time, the bacterial community structure and diversity of BSCs are highly susceptible to the combined influence of vegetation restoration type, the stage of crust development, physicochemical properties, and regional site conditions [16,17]. Compared to typical arid regions, the harsh water and heat conditions and poor ecosystem stability in alpine sandy land may result in certain specific patterns in the succession of BSCs.

Since most studies have not taken into account both the long-term effects of vegetation restoration and the characteristics of alpine sandy land, the response mechanisms of BSCs in different sand-fixing plantations remain unclear. This study proposes the following scientific hypothesis: under different sand-fixing plantations, the soil particle size distribution, physicochemical properties, enzyme activity, and bacterial communities of BSCs all exhibit regular changes with succession stages. Among these, physicochemical properties and enzyme activity are the key factors driving changes in bacterial communities. Samples of bare sand, algae crusts, and moss crusts were collected from beneath four types of plantations in alpine sandy land. A systematic analysis was conducted on soil particle size distribution, physicochemical properties, enzyme activity, bacterial community structure, and alpha diversity. This study aims to elucidate the succession patterns and key driving factors of BSCs. The results will provide a scientific basis and practical guidance for tree species selection and ecological management in afforestation projects in alpine sandy areas.

## 2. Materials and Methods

### 2.1. Study Area Description

**Figure 1:**
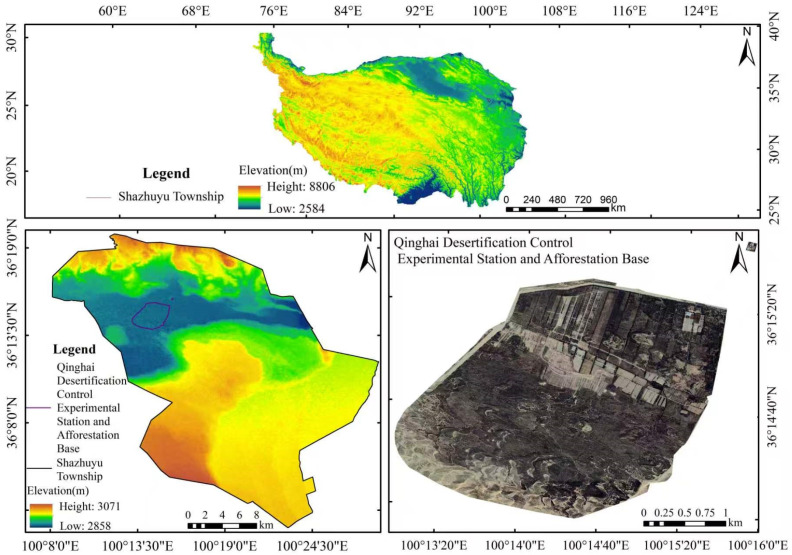
Location of the study area. Note: Base maps include 90 m DEM [18], 0.5 m WorldView-2 imagery and public township map data.

The study area is located in the Shazhuyu region of the Gonghe Basin, Hainan Prefecture, Qinghai Province, on the northeastern Tibetan Plateau. It is one of the most severely desertified regions in Qinghai Province, extending approximately 66 km east–west and 72 km north–south (99°45′–100°30′ E, 36°03′–36°40′ N). Elevations range from 2800 to 3100 m. The mean annual temperature is 4.1 °C, and the mean annual precipitation varies between 250 and 450 mm. The region features a typical high-altitude continental climate with cold, arid conditions and low precipitation, accompanied by severe desertification risks. After long-term ecological restoration, scattered forest patches have formed, and widely distributed sand-stabilizing shrubs and trees have promoted the extensive development of BSCs across this sandy ecosystem. The main vegetation includes *Populus cathayana*, *Populus simonii*, *Caragana microphylla*, *Salix psammophila*, *Caragana korshinskii*, and *Salix cheilophila*, among others. The location of the study area is provided below (Figure 1). The topographic background of the study area was generated using a 90 m Digital Elevation Model (DEM) from the National Tibetan Plateau Data Center [18]. Township map data was derived from public geographic datasets. The high-resolution satellite image of the experimental site was provided by the Qinghai Desertification Control Experimental Station and Afforestation Base was derived from WorldView-2 imagery with a panchromatic resolution of 0.50 m.

### 2.2. Collection of BSC Samples

In this study, we selected four sand-fixing plantation plots (*Salix psammophila*, SL; *Caragana korshinskii*, NT; *Salix cheilophila*, WL; *Populus simonii*, XYY) established in 1990. Algal and moss crusts were widely distributed across the plantations, while other crust types had extremely low coverage. Therefore, this study focused solely on algal and moss crusts. Algal crusts represent the early successional stage of BSCs in alpine sandy land. They have a thickness of 1 to 3 mm. They appear dark green, blackish brown, or yellowish brown. These crusts are thin, fragile, and discontinuous. Moss crusts are the late successional type. They develop on mature algae crusts. Their thickness ranges from 5 to 12 mm. When moist, they form continuous layers in dark green. They also bind tightly to the topsoil. Algae crusts mainly belong to Cyanophyta, Chlorophyta and Bacillariophyta, while all moss crusts are classified into Bryopsida [19].

Sampling was conducted in early to mid-October 2025 (This period is the optimal growth time for BSCs). In each plot, three large quadrats of 60 m × 60 m and six small quadrats of 6 m × 6 m were randomly established (to calculate the coverage and thickness of different types of crusts). Using random and multi-point sampling methods, bare sand (no crust formation, 0–2 cm soil layer), algae crusts, and moss crusts were collected from each large plot. The samples from each plot were combined into a composite soil sample, yielding a total of 36 soil samples. Before sampling, the shovel was disinfected with alcohol. After collection, the samples were placed in sterile resealable bags and labeled. GPS was used to record field coordinates, elevation, vegetation types, and other information for each sampling site. Each soil sample was divided into two portions: one portion was air-dried naturally for the determination of soil physicochemical properties, whereas the other was stored in an ice pack and transported to the laboratory for bacterial community analysis. Basic information on the samples is provided in the table below (Table 1).

### 2.3. Determination of the Physicochemical Properties and Enzyme Activities of BSCs

Soil particle size distribution was determined using a Mastersizer 2000 laser particle size analyzer (Malvern Instruments Ltd., Malvern, Worcestershire, UK). Optical parameters, dispersion conditions and obscuration were set prior to sample loading. Analysis was initiated once all parameters met the requirements [20]. A soil suspension (soil:water = 1:2.5) was allowed to settle, and the pH of the supernatant was measured with a pH meter (Sartorius AG, Goettingen, Germany). The moisture content of air-dried and fresh soil samples was determined by the oven-drying method with parallel tests, and soil water content (SWC) was calculated accordingly [21]. The alkali hydrolysis diffusion method was used. Soil was hydrolyzed with 1 mol/L NaOH (Shanghai, China). The released NH_3_ (Shanghai, China) was absorbed by boric acid. Then, the soil was titrated with a 0.005 mol/L H_2_SO_4_ (Shanghai, China) standard solution to determine the alkali-hydrolyzable nitrogen (AN) content [21]. Soil samples were digested in an oil bath using the external heating method with potassium dichromate and concentrated sulfuric acid, and the remaining K_2_Cr_2_O_7_ (Shanghai, China) was titrated with a FeSO_4_ (Shanghai, China) standard solution, and soil organic matter (SOM) content was calculated based on blank tests and correction factors [21]. After grinding and sieving, soil samples were weighed and encapsulated. Total carbon (TC) and total nitrogen (TN) were automatically quantified with a FlashSMART elemental analyzer (Thermo Fisher Scientific, Waltham, MA, USA) through inert gas purging, high-temperature combustion-reduction, and gas separation, using the calibration curve for calculation. Soil available phosphorus (AP) was determined by the sodium bicarbonate extraction–molybdenum-antimony colorimetric method. After isothermal shaking, filtration and color development, spectrophotometric measurement was conducted against the standard curve [21]. Soil available potassium (AK) was extracted by isothermal shaking with 1.00 mol/L CH_3_COONH_4_ ( Shanghai, China) solution. The filtrate was analyzed using a flame photometer. A standard curve was prepared with potassium standard solutions, and AK content was calculated subsequently [21]. Soil catalase (CAT) activity was assayed by potassium permanganate titration. Samples reacted with H_2_O_2_ (Shanghai, China) at a constant temperature, and residual hydrogen peroxide was titrated with a standard KMnO_4_ (Shanghai, China) solution. CAT activity was calculated according to the volume of titrant consumed [22]. Soil urease activity (URE) was measured using the sodium phenolate-sodium hypochlorite colorimetric method. Following incubation and color development, absorbance was recorded at 578 nm, and URE was calculated using the standard curve [23]. For the determination of soil alkaline phosphatase (ALP) activity, samples were incubated and developed color via the disodium hydrogen phosphate colorimetric method. Absorbance was measured at 660 nm, and ALP activity was calculated based on the standard curve [24]. The filtrate was mixed with 3,5-dinitrosalicylic acid (Shanghai, China) for color development in a boiling water bath, then cooled and diluted to a fixed volume. Absorbance was detected at 508 nm, and soil sucrase (SUC) activity was calculated from the measured values [25].

### 2.4. Species Identification, DNA Extraction, and Bacterial Community Analysis

Algae and moss crusts were identified via morphological observation. Algae crusts were identified to the phylum level using an optical microscope (Carl Zeiss AG, Oberkochen, Germany), while moss crusts were distinguished based on morphology and tissue structure. Soil samples of the two crust types were resuspended in sterile phosphate-buffered (Shanghai, China) saline and subjected to low-speed centrifugation to separate algae and moss tissues from soil bacteria. Sediment and supernatant were collected separately for nucleic acid extraction. Algae, moss crusts, and soil bacteria were analyzed individually: morphological analysis was performed for algae and moss crusts, and 16S rRNA high-throughput sequencing was applied for soil bacteria. This approach effectively differentiated their community characteristics.

Total bacterial DNA extraction and high-throughput sequencing were performed by Shanghai Majorbio Bio-Pharm Technology Co., Ltd. (Shanghai, China). Total bacterial DNA was extracted from soil samples using the MagaBio Soil Genomic DNA Purification Kit (Omega Bio-tek, Norcross, GA, USA). DNA integrity was assessed by 1% agarose gel (Shanghai, China) electrophoresis, and DNA concentration and purity were determined using a NanoDrop 2000 UV–Vis spectrophotometer (Thermo Fisher Scientific, Waltham, MA, USA). Polymerase Chain Reaction (PCR) amplification targeted the V3–V4 region of the bacterial 16S rRNA gene using universal primers 338F and 806R. The sequences were 338F (5′-ACTCCTACGGGAGGCAGCAG-3′) and 806R (5′-GGACTACHVGGGTWTCTAAT-3′). PCR amplification was performed under the following conditions: initial denaturation (95 °C, 3 min), followed by 27 cycles of denaturation (95 °C, 30 s), annealing (55 °C, 30 s), and extension (72 °C, 45 s) for 27 cycles; followed by a final extension (72 °C, 10 min) and storage at 4 °C. PCR products were analyzed by 2% agarose gel electrophoresis, and target fragments were purified using a magnetic bead purification kit. PCR products were quantified and normalized, after which Illumina (Illumina, Inc., San Diego, CA, USA) sequencing libraries were constructed and subjected to paired-end sequencing.

### 2.5. Data Processing

To test the scientific hypotheses of this study, the following three analytical objectives were defined: (1) to detect significant differences in soil particle composition, physicochemical properties, enzyme activities, and bacterial alpha diversity among the bare sand, algae crust, and moss crust stages under different plantations; (2) to visualize and test the overall differences in bacterial community structure among treatment groups; (3) to reveal the relationships between bacterial community structure and environmental factors (soil properties and enzyme activities). The specific data processing methods are as follows:

Alpha diversity analysis: Bacterial community alpha diversity was analyzed using Mothur software (version v.1.30.2, https://mothur.org/wiki/calculators/ (accessed on 16 April 2026)) [26,27]. SPSS 27.0 was then used to test for significant differences (ANOVA, LSD, *p* < 0.05). The tested variables included soil particle composition, physicochemical properties, enzyme activities, and bacterial diversity indices of the BSCs. Beta diversity analysis: Principal coordinate analysis (PCoA) based on Bray–Curtis distances was performed to visualize the overall differences in bacterial community structure among treatment groups. Analysis of similarities (ANOSIM) was then used to test the statistical significance of community differences between groups [28,29]. Analysis of the relationship between community and environmental factors: Spearman’s correlation analysis was used to evaluate the correlations between physicochemical properties/enzyme activities and the dominant bacterial phyla [30]. Redundancy analysis (RDA) was then performed to explore the associations between community composition and physicochemical properties/enzyme activities [31]. Furthermore, the Mantel’s test was used to analyze the overall correlation between community structure and all environmental factors [32]. All figures were plotted using R software (v3.3.1).

## 3. Results

### 3.1. Particle-Size Distribution of BSCs Under Different Sand-Fixing Plantations

**Figure 2:**
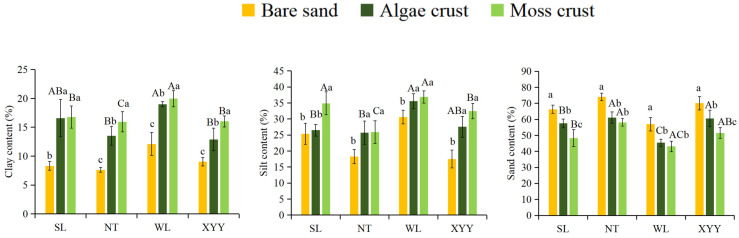
Particle size distribution of BSCs. Note: Different uppercase letters indicate significant differences (*p* < 0.05) among the same crust type across different plots, whereas different lowercase letters indicate significant differences (*p* < 0.05) in the parameters of different bark types within the same plot; SL: *Salix psammophila*; NT: *Caragana korshinskii*; WL: *Salix cheilophila*; XYY: *Populus simonii*.

The particle-size distribution of BSCs varied among the four types of sand-fixing plantations (Figure 2). The contents of silt and clay in BSCs under the four plantation types were significantly higher than those in bare sand, whereas sand content showed the opposite trend. As BSCs developed, the content of silt and clay particles gradually increased, while the sand content gradually decreased. Across the four plantation types, silt and clay contents followed the order: moss crust > algae crust > bare sand. Sand content followed the opposite order: bare sand > algae crust > moss crust. Overall, sand dominated the particle-size distribution of BSCs. It accounted for more than 40% of the total particle composition. In contrast, silt and clay contents were relatively low. Further comparison of grain-size composition at different developmental stages showed a clear pattern. Under WL plantations, both algae crusts and moss crusts had the highest silt and clay contents. These two crust types also had the lowest sand content under WL plantations.

### 3.2. Physicochemical Properties of BSCs Under Different Sand-Fixing Plantations

**Figure 3:**
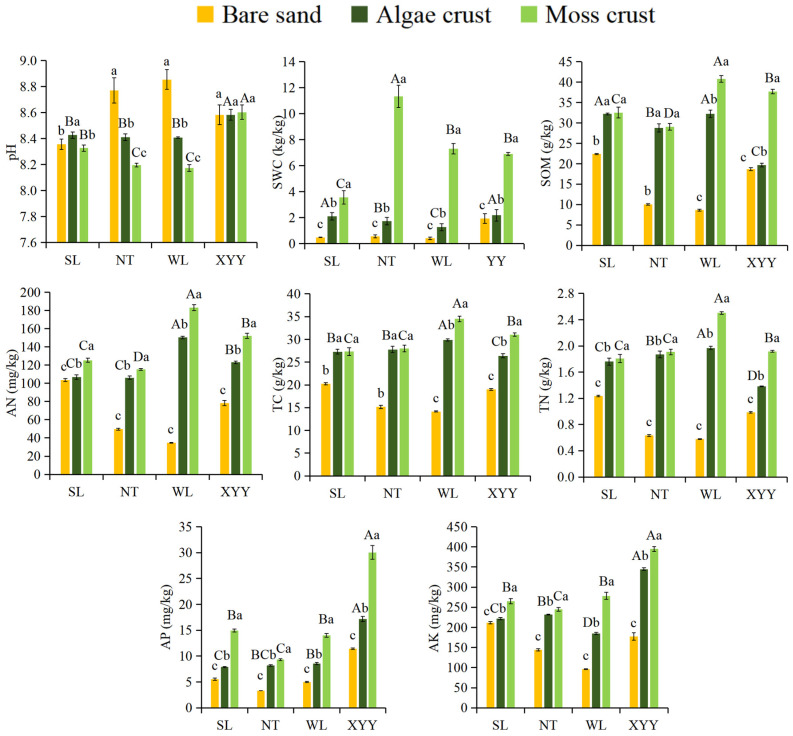
Physicochemical properties of BSCs. Note: Different uppercase letters indicate significant differences (*p* < 0.05) among the same crust type across different plots, whereas different lowercase letters indicate significant differences (*p* < 0.05) in the parameters of different bark types within the same plot; SL: *Salix psammophila*; NT: *Caragana korshinskii*; WL: *Salix cheilophila*; XYY: *Populus simonii*; SWC: soil water content; SOM: soil organic matter; AN: alkali-hydrolyzable nitrogen; TC: total carbon; TN: total nitrogen; AP: available phosphorus; AK: available potassium.

There are differences in the physicochemical properties of BSCs among the four types of sand-fixing plantations (Figure 3). As BSCs developed, the pH values were greater than 8 in all samples. This included bare sand, algae crusts, and moss crusts across all four sand-fixing plantations. These pH values indicate alkaline soil conditions. In the WL and NT plantations, the pH values of both crust types were significantly lower than those of bare sand. SWC gradually increased across all four plantation types, with SWC following the order: moss crust > algae crust > bare sand. Notably, SWC in moss crusts from the NT plantations was significantly higher than that in the other three plantation types. In terms of nutrient content, the levels of AN, TN, TC, SOM, AP, and AK in BSCs from all four plantations generally increased, with nutrients generally following the order: moss crust > algae crust > bare sand. Further comparison of nutrient contents at different stages of BSC development showed a clear pattern. In the WL plantations, both algae crust and moss crust had the highest AN, TC, TN, and SOM contents. In the XYY plantations, both crust types had the highest AP and AK contents.

### 3.3. Enzyme Activities of BSCs Under Different Sand-Fixing Plantations

**Figure 4:**
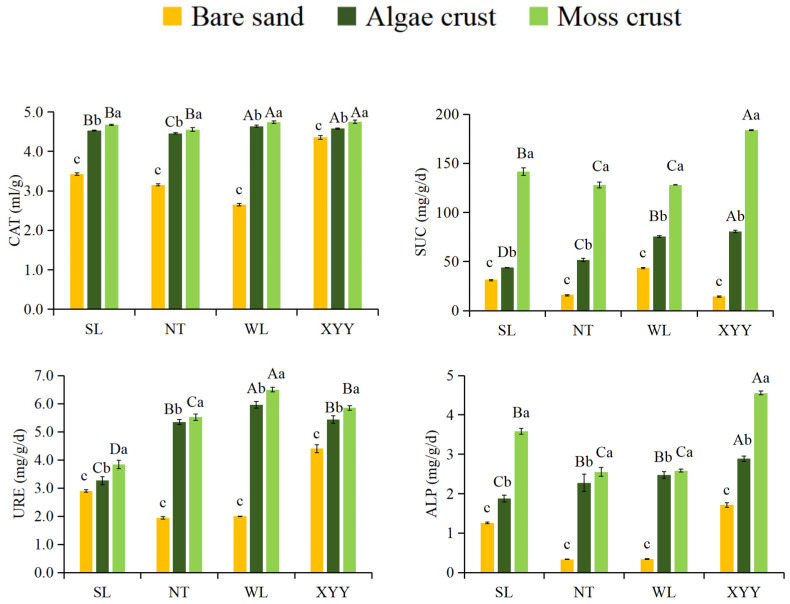
Enzyme activities of BSCs. Note: Different uppercase letters indicate significant differences (*p* < 0.05) among the same crust type across different plots, whereas different lowercase letters indicate significant differences (*p* < 0.05) in the parameters of different bark types within the same plot; SL: *Salix psammophila*; NT: *Caragana korshinskii*; WL: *Salix cheilophila*; XYY: *Populus simonii*; CAT: catalase; SUC: sucrase; URE: urease; ALP: alkaline phosphatase.

Enzyme activities differed among BSCs for all four types of sand-fixing plantations (Figure 4). In all four types of plantations, enzyme activities in bare sand were significantly lower than those in algae and moss crusts. As the BSCs developed, the activity of both types of lichenase in the four plantation species showed an upward trend, with the following order of activity: moss crust > algae crust > bare sand. Overall, the enzymatic activity of moss crusts is significantly higher than that of algae crusts. Further comparison of enzyme activities at different stages of BSC development showed a clear pattern. The activities of key BSC enzymes differed significantly among plantations. In the XYY plantations, CAT, SUC, and ALP activities were highest in both algae crust and moss crust. In the WL plantations, URE activity was highest in both crust types.

### 3.4. Alpha Diversity of Bacterial Communities in the BSCs Under Different Sand-Fixing Plantations

**Figure 5:**
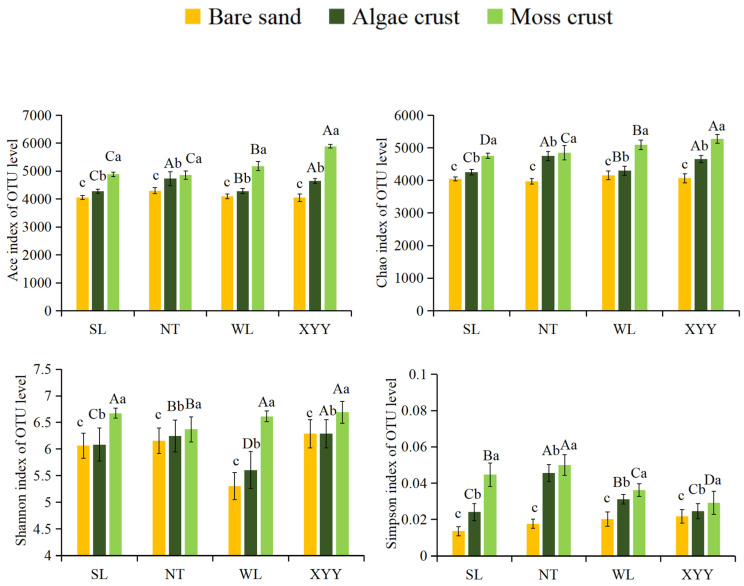
Alpha diversity of bacterial communities in BSCs. Note: Different uppercase letters indicate significant differences (*p* < 0.05) among the same crust type across different plots, whereas different lowercase letters indicate significant differences (*p* < 0.05) in the parameters of different bark types within the same plot; SL: *Salix psammophila*; NT: *Caragana korshinskii*; WL: *Salix cheilophila*; XYY: *Populus simonii*.

Alpha diversity is a key indicator of the complexity and stability of bacterial communities. Bacterial community richness and diversity were evaluated using alpha diversity indices in BSCs from four sand-fixing plantations (Figure 5). The ACE, Chao1 and Shannon indices across the four plantation types were generally high, whereas the Simpson index was generally low. Compared with bare sand, BSCs showed higher alpha diversity indices. As BSCs developed, all diversity indices generally increased, following the order: moss crust > algae crust > bare sand. Further comparison of diversity indices at different stages of BSC development showed a clear pattern. In the XYY plantations, the ACE, Chao1, and Shannon indices were highest. At the same time, the Simpson index was lowest in the XYY plantations. Specifically, bacterial communities in BSCs from XYY plantations showed the highest species richness. Their bacterial diversity was also significantly greater than that in the other plantations.

### 3.5. Bacterial Community Composition of BSCs in Different Sand-Fixing Plantations

**Figure 6:**
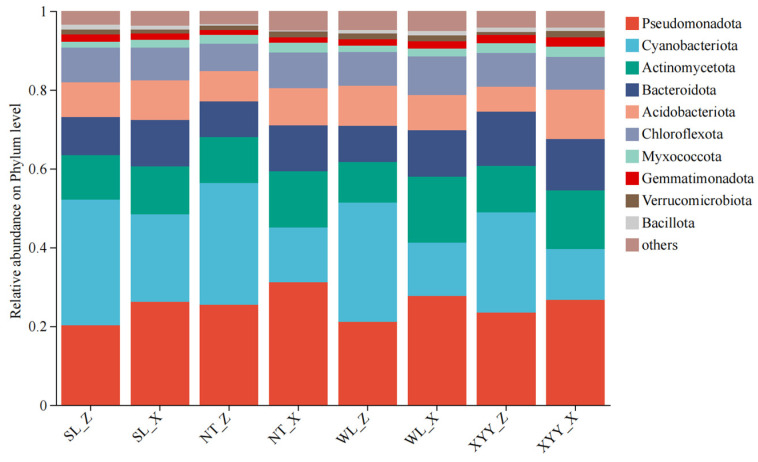
Bacterial community composition in BSCs from different plantations at the phylum level. Note: Only the top 10 most abundant bacterial phyla are shown; SL: *Salix psammophila*; NT: *Caragana korshinskii*; WL: *Salix cheilophila*; XYY: *Populus simonii*; Z: algae crust; X: moss crust.

A total of 10 bacterial phyla were identified in the BSCs of the four types of sand-fixing plantations (Figure 6). In all four plantations, the relative abundance of the dominant phylum Cyanobacteriota was significantly higher in algae than in moss crusts. Conversely, Pseudomonadota showed a significantly higher relative abundance in moss crusts compared with algae crusts within the same plantations. Cyanobacteriota was the most abundant phylum in algae crusts across all four plantations. Its relative abundances were 31.86%, 30.86%, 30.35%, and 25.42%, respectively. Pseudomonadota was the dominant phylum in moss crusts across all plantations. Its relative abundances were 26.27%, 31.19%, 27.70%, and 26.68%, respectively. Among bacterial phyla with relative abundances above 10%, the dominant phyla in algae crusts were Pseudomonadota, Cyanobacteriota, and Actinomycetota. Moss crusts additionally included Bacteroidota as a dominant phylum.

### 3.6. Differences in Bacterial Community Structure Among BSCs Under Different Plantations

**Figure 7:**
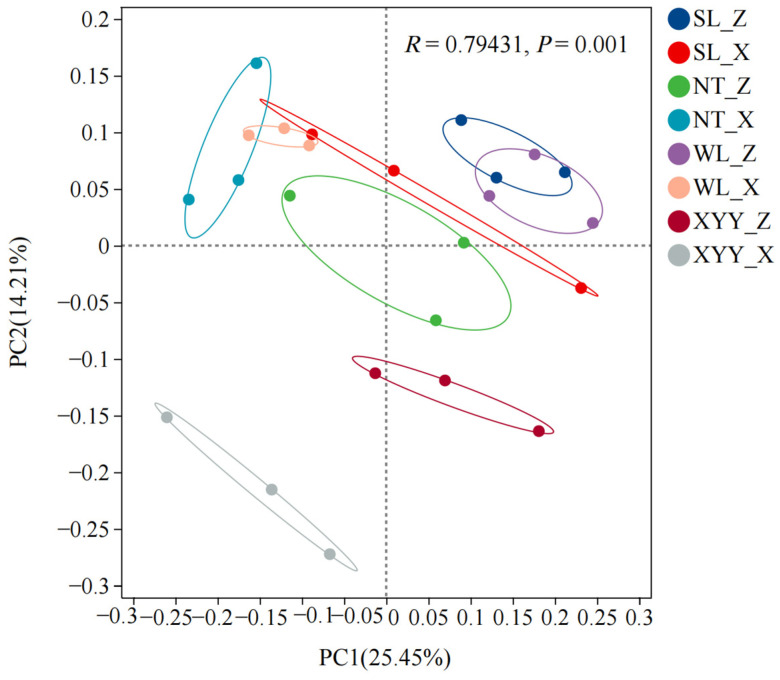
Principal Coordinate Analysis (PCoA) of bacterial community structure in BSCs from different plantations. Note: Similarity Analysis ANOSIM results showed (*R* = 0.79431 and *p* = 0.001); SL: *Salix psammophila*; NT: *Caragana korshinskii*; WL: *Salix cheilophila*; XYY: *Populus simonii*; Z: algae crust; X: moss crust.

Principal coordinate analysis (PCoA) was conducted at the OTU level (Figure 7). Principal coordinates first and second axes explained 25.45% and 14.21% of the variation, respectively. These two axes together accounted for 39.66% of the differences in community composition. Samples of algae and moss crusts from the same plantations showed varying degrees of overlap and separation, whereas samples from different plantations showed clear separation along the PC1 axis. Among these, the bacterial community in moss crusts of XYY plantations showed the greatest differentiation from the other groups. This indicates the highest community specificity. Similarity Analysis (ANOSIM) revealed significant effects on bacterial community structure (*R* = 0.79431, *p* = 0.001). These effects came from sand-fixing plantation types, BSC types, and their interaction. Specifically, highly significant differences (*p* < 0.01) were observed in bacterial communities between algae crusts from each plantation and moss crusts from the other plantations. Moreover, significant differences (*p* < 0.01) were observed between algae and moss crusts across all four plantations.

### 3.7. Correlation Among Physicochemical Properties, Enzyme Activities, and Bacterial Community Structure in Algae Crusts

**Figure 8:**
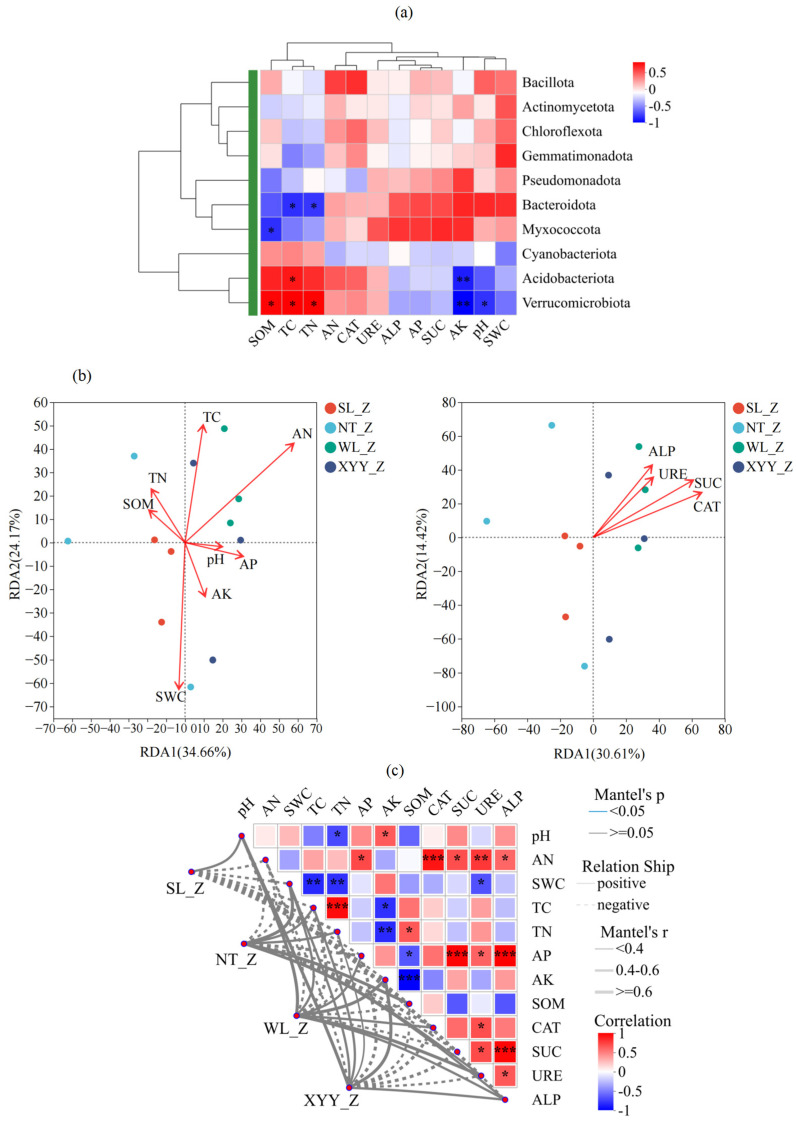
Correlation among physicochemical properties, enzyme activities, and bacterial community structure in algae crusts. Note: (**a**) Spearman’s correlation heatmap of physicochemical properties, enzyme activities, and major bacterial phyla in algae crusts; (**b**) redundancy analysis (RDA) plot of physicochemical properties, enzyme activities, and bacterial community structure in algae crusts; (**c**) Mantel’s test heatmap of physicochemical properties and enzyme activities of algae crusts; In the heatmaps, red indicates positive correlations, blue indicates negative correlations, and asterisks indicate significance levels: * 0.01 < *p* ≤ 0.05, ** 0.001 < *p* ≤ 0.01, *** *p* ≤ 0.001; SL: *Salix psammophila*; NT: *Caragana korshinskii*; WL: *Salix cheilophila*; XYY: *Populus simonii*; Z: algae crust; SWC: soil water content; SOM: soil organic matter; AN: alkali-hydrolyzable nitrogen; TC: total carbon; TN: total nitrogen; AP: available phosphorus; AK: available potassium; CAT: catalase; SUC: sucrase; URE: urease; ALP: alkaline phosphatase.

A correlation analysis was performed on the physicochemical properties, enzyme activities, and bacterial community structure of algae crust in four types of sand-fixing plantations (Figure 8). Spearman’s correlation analysis (Figure 8a) indicates that the physicochemical properties and enzyme activities of algae crusts had limited effects on the relative abundances of major bacterial phyla. TC is significantly positively correlated with Acidobacteriota; SOM, TC and TN are significantly positively correlated with Verrucomicrobiota. The redundancy analysis (RDA) results (Figure 8b) indicated that the physicochemical properties and enzyme activities of algae crusts significantly influence the bacterial community structure. The first and second axes explained 58.83% and 45.03% of the variation. This variation came from three types of variables in algae crusts. These were bacterial communities, physicochemical properties, and enzyme activities. The algae crusts were collected from the four types of plantations. AP, AN, SOM, and CAT are the key drivers regulating the community structure of bacteria in algae crusts across four types of plantations. Mantel’s tests further indicate (Figure 8c) that the algae crusts on these four plantations show a significant correlation with soil factors and bacterial communities. In SL plantations, the bacterial community correlated positively only with pH; in NT plantations, it correlated positively with TC, TN, AP, SOM, CAT, SUC, URE, and ALP; in WL plantations, positive correlations were found with pH, TC, TN, AP, AK, CAT, SUC, URE, and ALP; in XYY plantations, positive correlations were found with pH, AN, TC, TN, AP, AK, SOM, CAT, SUC, URE, and ALP.

### 3.8. Correlation Among Physicochemical Properties, Enzyme Activities, and Bacterial Community Structure in Moss Crusts

**Figure 9:**
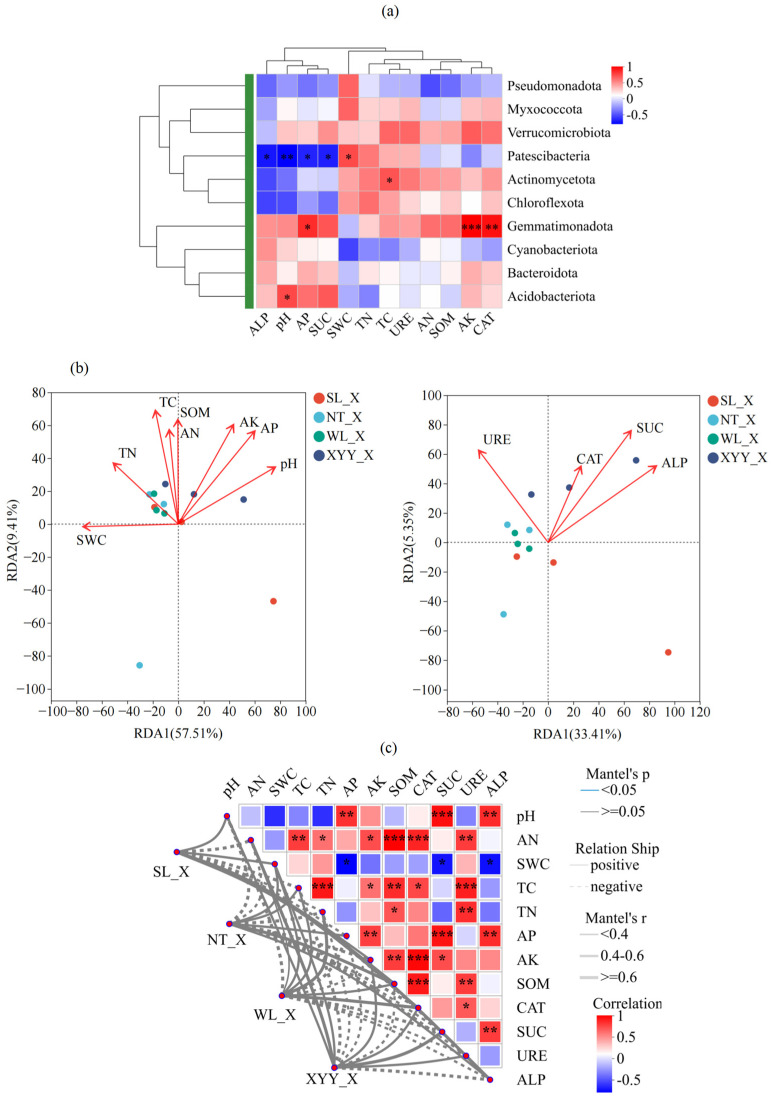
Correlation among physicochemical properties, enzyme activities, and bacterial community structure in moss crusts. Note: (**a**) Spearman’s correlation heatmap of physicochemical properties, enzyme activities, and major bacterial phyla in moss crusts; (**b**) redundancy analysis (RDA) plot of physicochemical properties, enzyme activities, and bacterial community structure in moss crusts; (**c**) Mantel’s test heatmap of physicochemical properties and enzyme activities of moss crusts; In the heatmaps, red indicates positive correlations, blue indicates negative correlations, and asterisks indicate significance levels: * 0.01 < *p* ≤ 0.05, ** 0.001 < *p* ≤ 0.01, *** *p* ≤ 0.001; SL: *Salix psammophila*; NT: *Caragana korshinskii*; WL: *Salix cheilophila*; XYY: *Populus simonii*; X: moss crust; SWC: soil water content; SOM: soil organic matter; AN: alkali-hydrolyzable nitrogen; TC: total carbon; TN: total nitrogen; AP: available phosphorus; AK: available potassium; CAT: catalase; SUC: sucrase; URE: urease; ALP: alkaline phosphatase.

Correlation analysis was conducted on moss crusts from four types of sand-fixing plantations (Figure 9). The analysis included physicochemical properties, enzyme activities, and bacterial community structure. The results showed a clear pattern. Compared with algae crusts, moss crusts had stronger correlations. Specifically, their physicochemical properties and enzyme activities correlated more strongly with the relative abundances of major bacterial phyla. Spearman’s correlation analysis showed (Figure 9a) that pH was significantly positively correlated with Gemmatimonadota and Acidobacteriota; SUC was significantly positively correlated with Patescibacteria; TC was significantly positively correlated with Actinomycetota; AK and CAT were significantly positively correlated with Gemmatimonadota; and ALP, pH, AP, and SUC were significantly negatively correlated with Patescibacteria. The redundancy analysis (RDA) results (Figure 9b) indicated that soil physicochemical properties and enzyme activities significantly influenced the bacterial community structure of moss crusts. The first and second axes explained 66.92% and 38.76% of the total variation in bacterial communities, environmental factors, and enzyme activities in moss crusts from the four plantations. AP, pH, SWC, and ALP are the key drivers shaping the community structure of bacteria in BSCs across four types of plantations. Mantel’s tests also showed (Figure 9c) that bacterial communities in moss crusts from the four plantations were significantly correlated with both soil physicochemical properties and enzyme activities. Specifically, in SL plantations, the bacterial community correlated positively with pH and TC; in NT plantations, the bacterial community correlated positively only with TC, AP, SOM, CAT, SUC, and ALP; in WL plantations, the bacterial community correlated positively only with TC, TN, AK, SOM, and CAT; in XYY plantations, the bacterial community correlated positively only with pH, AN, TC, AP, AK, SOM, CAT, SUC, URE, and ALP.

## 4. Discussion

### 4.1. Differences in Particle Composition, Physicochemical Properties, and Enzyme Activities of BSCs Under Different Sand-Fixing Plantations

BSCs are an important component of desert ecosystems and a key pioneer group for the ecological restoration of degraded sandy lands. Numerous studies have shown that BSCs can, to a certain extent, alter soil physicochemical properties and enzyme activities, thereby promoting nutrient cycling and contributing to windbreak and sand fixation, curbing desertification, and restoring ecosystems [33,34]. However, owing to the heterogeneity of regional habitat conditions, there are significant differences in the physicochemical properties and bacterial community characteristics of BSCs among different study areas. Differences were observed in the particle size distribution, physicochemical properties, and enzyme activities of BSCs from different types of sand-fixing plantations. The results of the grain size analysis show that the content of silt and clay particles in the BSCs from the four plantations was significantly higher than that in bare sand, whereas sand content showed the opposite trend. This indicates that BSCs exert a soil-refining effect, significantly increasing the content of fine soil particles [35,36,37]. As BSCs developed, the proportion of fine particles in the four plantation types followed the order of moss crust > algae crust > bare sand. This indicates that BSCs can significantly improve crusted soils by promoting the accumulation of fine particles and improving soil structure [38,39]. Among these, BSCs from the WL plantations had the highest fine particle content. This is consistent with the findings of Zhang et al. [35,36,37]. This may be related to the windbreak effect of the WL plantations canopy and the formation of a protective layer, resulting in finer soil particles [40].

The results of the physicochemical properties analysis show that the bare sand, algae crusts, and moss crusts in all four plantations were alkaline (pH > 8). This is consistent with the alkaline nature of sandy soils [41,42]. The pH values of the BSCs in the WL and NT plantations were both significantly lower than those of the bare sand. The results indicate that BSCs in WL and NT plantations can reduce soil alkalization [38,39]. The SWC of BSCs in the four types of plantations consistently followed the order: moss crust > algae crust > bare sand. This indicates that BSCs have a strong water-holding capacity and can inhibit soil moisture evaporation [43]. Among them, the moisture content of moss crusts in the NT plantations was significantly higher than in the other three types of plantations. This may be related to the high saturated SWC, total porosity, and high water-holding capacity of the moss crusts [44]. As the BSCs developed, the nutrient contents of AN, TN, TC, SOM, AP, and AK generally showed an increasing trend, and nutrient levels in bare sand were significantly lower than those in algae and moss crusts. This is consistent with the findings of Guo et al. [45] and Du et al. [41,42]. A further comparison of nutrient contents in BSCs at different developmental stages revealed clear patterns. In WL plantations, AN, TC, TN, and SOM levels were highest. In XYY plantations, AP and AK levels were highest. This difference may be related to the greater capacity of WL plantations to accumulate organic matter and retain nutrients in the BSCs [35,36,37]. This is related to the fact that BSCs in XYY plantations are more effective at activating readily available phosphorus and potassium and supplying available nutrients [46]. The enzyme activity results showed that the enzyme activities in the four plantations were significantly lower than those in algae and moss crusts. This difference may be associated with microbial community complexity, nutrient levels, and moisture conditions [35,36,37]. As BSCs developed, the activity of all four enzymes followed the order: moss crust > algae crust > bare sand. It has been shown that the development and succession of BSCs can significantly increase enzyme activities in the crust layer [47]. A further comparison of enzyme activities in BSCs at different developmental stages showed clear patterns. In XYY plantations, CAT, SUC, and ALP activities were highest. In WL plantations, URE activity was highest. This variation may be driven by factors such as extracellular proteins in BSCs, the carbon-to-nitrogen (C/N) ratio, and total phosphorus (TP) [48].

### 4.2. Differences in Bacterial Community Structure of BSCs Among Different Sand-Fixing Plantations

Bacterial communities are important indicators of ecosystem function. This study found that the α-diversity, composition, and structure of bacterial communities in BSCs varied among different types of sand-fixing plantations. The bacterial community α-diversity results showed that the Ace, Chao1, Shannon, and Simpson indices for BSCs from the four plantation types were all higher than those for bare sand. This is consistent with the findings of Li et al. [49] in the Mu Us Desert. As BSCs developed, the diversity indices showed the following order: moss crust > algae crust > bare sand. Findings indicate that BSCs promote an increase in soil microbial community diversity [50]. When comparing the diversity indices of BSCs at different developmental stages, the Ace, Chao1, and Shannon indices were all highest in the XYY plantations, while the Simpson index was lowest in the XYY plantations. This indicates that the bacterial communities in the XYY plantations are species-rich and highly diverse. This likely occurs because vegetation improves the BSC microenvironment by altering nutrient levels, releasing root exudates, and increasing litterfall input. This, in turn, provides resources for a wider range of bacteria, thereby enhancing bacterial richness and diversity [38,39,51].

Microbial community analysis revealed that Cyanobacteriota was the dominant phylum in algae crusts, whereas Pseudomonadota dominated in moss crusts. This is consistent with the findings of Jiao et al. [52]. This indicates that algae crusts rely more on the nitrogen-fixing function of Cyanobacteriota, whereas moss crusts depend more on the nutrient transformation function of Pseudomonadota [53]. This may be related to the fact that the Cyanobacteriota, as a key functional group in algae crust formation, drives soil carbon and nitrogen cycles and ecosystem stability through processes such as photosynthetic carbon fixation and biological nitrogen fixation [17,54]. The Pseudomonadota, as a key functional group within the moss crust, are central drivers of soil carbon and nitrogen cycles. Meanwhile, for the algae and moss crusts of the four sand-fixing plantations, the dominant Cyanobacteriota and Pseudomonadota exhibited an obvious competitive succession pattern with BSC development, showing a clear trade-off in their predominance. Additionally, compared with algae crusts (relative abundance > 10%), moss crusts were further enriched with additional such as Bacteroidota. This indicates that the succession of BSCs is not merely a change in morphological appearance but also a process of microbial community structure remodeling and gradual optimization of ecological functions [55,56].

The PCoA and ANOSIM results indicate that there are highly significant differences in bacterial community structure between algae and moss crusts in the same plantations. This is consistent with the findings of Zhang et al. [35,36,37] and Liu et al. [50]. This indicates that the development of BSCs and sand-fixing plantation types is an important factor driving the succession of dominant bacterial groups [57,58]. Among these, the microbial community in the moss crust of the XYY plantations showed the greatest differences from the other groups and exhibited the highest community specificity. Compared with other plantations, this may be due to the strong directional selection effect of litter quality, rhizosphere secretions, and nutrient characteristics of the XYY plantations on the microbial community [58].

### 4.3. Differential Analysis of Correlations Between Physicochemical Properties, Enzyme Activities, and Bacterial Communities of BSCs in Different Sand-Fixing Plantations

This study also found that the correlations between the physicochemical properties and enzyme activities of BSCs from different sand-fixing plantations and bacterial communities varied. Spearman’s correlation analysis revealed significant positive and negative correlations among BSCs from the four types of plantations regarding soil physicochemical properties, enzyme activities, and the relative abundance of dominant bacterial phyla. This study demonstrates that the physicochemical properties and enzyme activities of BSCs influence the composition of bacterial communities [35,36,37,59]. However, compared with algae crusts, the physicochemical properties and enzymatic activities of moss crusts show stronger correlations with the relative abundance of major bacterial phyla. This difference is associated with higher nutrient accumulation and greater enzyme activities in the moss crust, as well as a more complex bacterial community structure [58,60]. The RDA results indicate that SWC is a key physicochemical factor influencing bacterial communities in moss crusts from the four plantations. This demonstrates that different forest stands drive the positive succession of BSC-associated bacterial communities by regulating soil water retention capacity [11]. CAT and ALP activities had the most significant effects on the bacterial communities associated with algae and moss crusts, respectively, in the four plantations. This indicates that the primary metabolic functions of the soil bacterial community undergo a functional shift during the development of BSCs [59]. The results of the Mantel’s test indicate that the environmental drivers of bacterial communities in BSCs at different developmental stages exhibit distinct patterns of differentiation. This is consistent with the findings of Liu et al. [58]. AP and carbon/phosphorus cycle-related enzyme activities (CAT, SUC, URE) were common key drivers across the four plantation types. In contrast, factors such as pH, SWC, AK, SOM, and ALP showed strong plantation-specific effects. This indicates that the activity of enzymes involved in the carbon and phosphorus cycles plays a key regulatory role in the bacterial communities associated with algae blooms [61]. In moss crusts, TC, SOM, and AP served as common drivers of bacterial communities across the four sand-fixing plantations, whereas other factors acted as plantation-specific drivers with significant variability. This indicates that moss crusts accumulate abundant nutrients due to their high level of development and have a stable microenvironment [60]. The findings clarify the mediating role of soil physicochemical properties and enzyme activities in regulating bacterial community structure during the developmental stages of BSCs [62].

In summary, based on 30+ years of succession data from BSCs in sand-fixing plantations established in 1990: WL plantations are recommended for wind erosion control and long-term accumulation of nitrogen and organic matter; XYY plantations are preferred for alleviating phosphorus and potassium limitation and enhancing soil enzyme activities. The moss crust bacterial community under XYY serves as a 30-year baseline for assessing future environmental disturbances. Furthermore, regulating soil factors such as pH and water content can accelerate algae-to-moss crust succession, and selecting XYY for new plantations maximizes long-term soil microbial diversity. These findings indicate that the differences in bacterial communities within BSCs across various sand-fixing plantations in alpine sandy land are driven by multiple interacting factors. Physicochemical properties and enzyme activities act as the key direct drivers of BSC bacterial community structure, while stand type and crust succession exert indirect influences. These results also reflect the adaptive strategies of bacteria to high-altitude environments and the shaping role of environmental filters in structuring bacterial communities. Root exudates and litter effects were not investigated here, representing potential directions for future work.

## 5. Conclusions

(1) As BSCs developed, the proportion of fine soil particles (silt and clay) gradually increased, while the sand content gradually decreased. Among these, BSCs in WL plantations had the most pronounced effect on soil fine-graining and texture improvement.
(2) As BSCs developed, SWC, nutrient content, and enzyme activities generally increased. Specifically, BSCs in the WL plantations were more conducive to the accumulation of AN, TC, TN, and SOM, as well as increased URE activity, whereas BSCs in the XYY plantations were more conducive to the accumulation of AK and AP, as well as increased CAT, SUC, and ALP activity.
(3) The dominant bacterial phyla in BSCs from the study area were Pseudomonadota, Cyanobacteriota, Actinomycetota, and Bacteroidota. As BSCs developed, the richness and diversity of bacterial communities generally increased. Among them, the bacterial communities in the XYY plantations were rich and highly diverse, and the bacterial communities in their moss crusts differed most significantly from those in other plantations and exhibited the highest community specificity.
(4) AP, AN, SOM, and CAT are the key physicochemical factors influencing the bacterial community structure of algae crusts. AP, pH, SWC, and ALP are the key physicochemical factors influencing the bacterial community structure of moss crusts. In summary, physicochemical properties and enzyme activities act as the key direct drivers of BSC bacterial community structure, while stand type and crust succession exert indirect influences.

## Figures and Tables

**Table 1:** Basic sample information.

No.	Sample Plot	Sample Abbreviation	Crust Type	Crust Number	Crust Thickness (mm)	Crust Coverage (%)
1	*Salix psammophila*	SL	Bare sand	SL_W	-	-
			algae crust	SL_Z	5.89 ± 1.13	16.12 ± 6.10
			Moss crust	SL_X	7.42 ± 1.36	29.61 ± 7.45
2	*Caragana korshinskii*	NT	Bare sand	NT_W	-	-
			algae crust	NT_Z	5.75 ± 0.85	18.46 ± 3.02
			Moss crust	NT_X	10.16 ± 1.61	35.05 ± 4.33
3	*Salix cheilophila*	WL	Bare sand	WL_W	-	-
			algae crust	WL_Z	7.74 ± 0.36	31.72 ± 4.13
			Moss crust	WL_X	12.21 ± 1.43	40.25 ± 10.74
4	*Populus simonii*	XYY	Bare sand	XYY_W	-	-
			algae crust	XYY_W	7.15 ± 1.09	30.62 ± 12.38
			Moss crust	XYY_W	12.14 ± 3.12	39.39 ± 7.19

Note: Values are presented as mean ± standard deviation; “-” indicates not applicable; SL: *Salix psammophila*; NT: *Caragana korshinskii*; WL: *Salix cheilophila*; XYY: *Populus simonii;* W: bare sand; Z: algae crust; X: moss crust.

## Data Availability

The original contributions presented in this study are included in the article. Further inquiries can be directed to the corresponding authors.

## Abbreviations

The following abbreviations are used in this manuscript:

AK	available potassium
ALP	alkaline phosphatase
AN	alkali-hydrolyzable nitrogen
AP	available phosphorus
BSCs	biological soil crusts
CAT	catalase
PCoA	principal coordinates analysis
ANOSIM	Analysis of Similarities
RDA	redundancy analysis
SL	*Salix psammophila*
SOM	soil organic matter
SUC	sucrase
SWC	soil water content
TC	total carbon
TN	total nitrogen
URE	urease
WL	*Salix cheilophila*
XYY	*Populus simonii*
NT	*Caragana korshinskii*
PCR	Polymerase Chain Reaction
